# The Traffic Adaptive Data Dissemination (TrAD) Protocol for both Urban and Highway Scenarios

**DOI:** 10.3390/s16060920

**Published:** 2016-06-21

**Authors:** Bin Tian, Kun Mean Hou, Haiying Zhou

**Affiliations:** 1 LIMOS Laboratory CNRS UMR 6158, Blaise Pascal University, Clermont-Ferrand 63178, France; bin.tian@isima.fr; 2 School of Electrical & Information Engineering, HuBei University of Automotive Technology, Shiyan 442002, China; zhouhy_dy@huat.edu.cn

**Keywords:** VANETs, data dissemination, TrAD, broadcast storm suppression technique, store-carry-forward mechanism

## Abstract

The worldwide economic cost of road crashes and injuries is estimated to be US$518 billion per year and the annual congestion cost in France is estimated to be €5.9 billion. Vehicular Ad hoc Networks (VANETs) are one solution to improve transport features such as traffic safety, traffic jam and infotainment on wheels, where a great number of event-driven messages need to be disseminated in a timely way in a region of interest. In comparison with traditional wireless networks, VANETs have to consider the highly dynamic network topology and lossy links due to node mobility. Inter-Vehicle Communication (IVC) protocols are the keystone of VANETs. According to our survey, most of the proposed IVC protocols focus on either highway or urban scenarios, but not on both. Furthermore, too few protocols, considering both scenarios, can achieve high performance. In this paper, an infrastructure-less Traffic Adaptive data Dissemination (TrAD) protocol which takes into account road traffic and network traffic status for both highway and urban scenarios will be presented. TrAD has double broadcast suppression techniques and is designed to adapt efficiently to the irregular road topology. The performance of the TrAD protocol was evaluated quantitatively by means of realistic simulations taking into account different real road maps, traffic routes and vehicular densities. The obtained simulation results show that TrAD is more efficient in terms of packet delivery ratio, number of transmissions and delay in comparison with the performance of three well-known reference protocols. Moreover, TrAD can also tolerate a reasonable degree of GPS drift and still achieve efficient data dissemination.

## 1. Introduction

The economic impacts of road crashes, injuries and the annual congestion are very important, even without considering the impact on the environment [1,2]. Intelligent Transportation Systems (ITSs) integrate Vehicular Ad hoc Networks (VANETs) with an efficient Inter-Vehicle Communication (IVC) protocol and may help decrease significantly road crashes and road traffic congestion. In fact, VANETs can provide wireless communication technology among vehicles (V2V) or between vehicles and infrastructure (V2I) to support the development of various applications concerning traffic safety, transportation efficiency and infotainment on wheels [3]. Long range communication technologies have been used for most of the non-safety applications. In particular, cellular networks (3G/4G), associated with smartphones, have been investigated to satisfy the requirements of the respective applications [4,5]. In this paper, we focus on safety applications, in which the event-driven emergency messages, e.g., traffic accident warnings, must be efficiently disseminated within a specific geographical region [6]. High performance IVC protocols are the key for this requirement, namely data dissemination protocols. However, VANETs offers characteristics that are highly dynamic and data-intensive, spatially and temporally localized, which makes the design of data dissemination protocols a challenging task [7]. To solve these problems, in general a broadcast communication technique is often used to disseminate data packets because it is flexible enough to spread messages to vehicles with high dynamics in a region of interest (ROI) [8,9,10,11,12]. In addition, the applicability of disseminating data in both urban and highway scenarios has been proven and evaluated [10,11].

However, given the nature of vehicular networks and the short range communication issues, there are a number of challenges when designing routing protocols under the broadcast communication paradigm. First, the pure flooding scheme, a basic routing for broadcasts, can disseminate data into a wide area with low delay, but if the flooding is not properly controlled , a so-called “broadcast storm” may occur in dense networks [13]. The relaying scheme proposes selecting appropriate forwarders to mitigate this problem. Unfortunately, most relaying schemes focus exclusively on the highway or the urban scenario, therefore, how to select suitable forwarders in both highway and urban scenarios is a key open issue. Secondly, the distribution of vehicles suffers a large variability due to the high mobility of vehicles. Moreover, in urban scenarios the line-of-sight signal could be blocked because of the existence of buildings, consequently, the network links are not reliable. Thus, how to efficiently manage the connectivity between these separate vehicular networks is a critical challenge. In addition, the low proportion of Dedicated Short Range Communication (DSRC)-equipped vehicles in the initial market penetration phase is another reason that leads to the above issue. Finally, from a standards point of view, the IEEE 802.11p standard does not establish a Basic Service Set (BSS), which means it is not necessary to perform authentication and association procedures. Furthermore, the Carrier Sense Multiple Access with Collision Avoidance (CSMA/CA) mechanism of IEEE 802.11p does not have an acknowledgment process. These amendments indeed improve the speed of connection process, but lead to unreliable broadcasting connectivity.

In this paper, the Traffic Adaptive data Dissemination (TrAD) protocol is proposed in an attempt to address these problems in both urban and highway scenarios, while simultaneously aiming for high packet delivery ratio, low network overhead and low delay. The main contributions of this paper can be summarized as follows:

- For the broadcast suppression technique, a vehicular cluster classification and a sorting technique were designed to improve the reliability of broadcast transmissions and suppress the broadcast storm problem.
- For the store-carry-forward mechanism, specific vehicles were selected to be data-ferries so as to exchange information between different disconnected networks. Moreover, the redundant transmissions of this process were *further* suppressed.
- A channel congestion control was designed according to the status of local network traffic.
- A comprehensive performance evaluation was performed by means of realistic simulations. Three classical data dissemination protocols were compared with TrAD in both urban and highway scenarios. Furthermore, the impact of GPS drift on the data dissemination speed was also investigated.

The remainder of this paper is organized as follows. In Section 2, the state-of-the-art in data dissemination protocols are summarized and the issues in need of solutions are highlighted. Section 3 presents the TrAD protocol motivated by these challenges, and where every detailed technique or mechanism is elaborated. In Section 4, the performance of the TrAD protocol is evaluated by considering realistic scenarios. In the last section, we establish an assessment of the TrAD protocol and present the future work.

## 2. Related Work

Nowadays, in general, the works of most of data dissemination protocols focus on two main problems. The first challenge is the broadcast storm problem in well-connected networks; the second challenge is the disconnected network (network link reliability) or network partition problem. The broadcast suppression technique is adopted to mitigate the former issue and the store-carry-forward mechanism is designed for the latter one.

### 2.1. Broadcast Suppression Technique

Tseng *et al.* [13] first described the broadcast storm problem, and then proposed three pioneering schemes to mitigate it, *i.e.*, a *counter-based scheme*, *distance-based scheme* and *location-based scheme*. However, all these schemes are specified for Mobile Ad hoc Networks (MANETs) and do not consider the nature of VANETs. Wisitpongphan and Tonguz *et al.* [14] proposed three interesting and influential techniques for VANETs, *i.e.*, *weighted p-persistence*, *slotted 1-persistence*, and *slotted p-persistence*. After that, they went on to propose the Distributed Vehicular BroadCAST (DV-CAST) protocol that resorts to three flags that can provide the necessary knowledge about the local topology to identify the vehicles in well-connected networks. Among these vehicles, the authors recommended the use of the weighted p-persistence technique to suppress broadcast storms [8]. The slotted 1-persistence technique was improved by Schwartz *et al.* [15] for their Simple and Robust Dissemination (SRD) protocol. The optimized slotted 1-persistence defined different priorities in the two directions of the highway. In addition, the Emergency Message Dissemination for Vehicular (EMDV) environments protocol was proposed by Torrent-Moreno *et al.* [16] to improve the reliability of transmissions by using a forwarding area and a contention scheme. However, all the aforementioned efforts focus on one-dimensional highway scenarios.

For two-dimensional urban scenarios, Korkmaz *et al.* [17] proposed the so-called Urban Multi-hop Broadcast (UMB) protocol to address the broadcast storm, hidden node and reliability problems. UMB uses a Request to Broadcast (RTB) and Clear to Broadcast (CTB) handshake mechanism to select the furthest vehicle in a road segment to forward the packet. Moreover, UMB resorts to *repeaters* located at intersections to initialize a new broadcast according to the final destination. However, the deployment of repeaters will cost enormous financial and human resources, which is the chief stumbling block to implementing the protocol. Fogue *et al.* [18] proposed the enhanced Message Dissemination based on Roadmaps (eMDR) protocol that uses a real urban map and GPS information to identify the location of vehicles to more efficiently perform the data dissemination. The authors of DV-CAST also proposed a broadcast protocol for urban scenarios, Viriyasitavat and Tonguz *et al.* [9] proposed the Urban Vehicular BroadCAST (UV-CAST) protocol where the wait time to rebroadcast is calculated in terms of the location and the distance. A shorter rebroadcast delay is assigned to the vehicles located at an intersection, which intend to disseminate data messages in more directions. Indeed, Fogue *et al.* [19] highlighted that eMDR and UV-CAST should not blindly trust the GPS information that is usually not accurate. Thus, it is important to quantify the impact of GPS drift or error on the performance of IVC protocols.

The Data dissemination pRotocol In VEhicular networks (DRIVE) and Adaptive Multi-directional data Dissemination (AMD) protocols are able to classify neighbors into different quadrants or sectors, which can be used to disseminate data in both urban and highway scenarios [10,11]. DRIVE partitions the communication area into four quadrants and chooses a sub-area inside each quadrant called *sweet spot*. The vehicles located in the sweet spot have a shorter rebroadcast delay. AMD adaptively divides the vehicle’s communication area into two or four equal sectors according to the road topology and the distribution of neighboring vehicles. In fact, in the real world, particularly in European downtowns, the road topology is not always regular as the Manhattan grid-type one. The typical example is the road topology of the “Arc de Triomphe” in Paris, which has twelve roads gathered at a roundabout. The approaches of these two protocols are not flexible enough to recognize the irregular road topology.

### 2.2. Store-Carry-Forward Mechanism

The disconnected network or network partition problem has been extensively investigated in Delay Tolerant Networks (DTNs) to exploit opportunities to complement intermittent connections among mobile devices [20,21]. However, the data dissemination in VANETs has its own characteristics. The connection of vehicles is easily interrupted due to the high mobility of the vehicles themselves, the building obstacles, the interference and the short range communication [22,23]. The goal of the data dissemination is to efficiently inform all vehicles in a ROI about event-driven messages such as emergency messages. Therefore, the Store-Carry-Forward (SCF) mechanism not only needs to organize packets to be exchanged by an end-to-end paradigm in the one-hop range, but also to discover opportunities to start a new dissemination process in a further vehicular network.

Most of literature about the SCF mechanism investigated the selection of *SCF-agent* vehicles that can store and carry the data until they encounter new opportunities to forward it. DV-CAST can recognize two types of vehicles to be selected as the SCF-agent in highway scenarios. One is the last vehicle of a cluster driving in the same direction as the source vehicle, the other is the first vehicle of a cluster moving against the source vehicle [8]. UV-CAST used a gift-wrapping algorithm to select all boundary vehicles to be the SCF-agent. It is to be noted that this algorithm is a distributed version, and the set of SCF-agent vehicles is always a superset of all boundary vehicles. Therefore, UV-CAST would trigger more redundant transmissions. DRIVE did not resort to beaconing, but it only used the vehicles outside the ROI as information ferries to handle the network partition problem. It is obvious that most transmission opportunities would be missed. AMD did not suffer this problem, and it used beaconing to determine whether a vehicle is a *tail* or not. If a vehicle makes a transition from *tail* to *non-tail*, a new broadcast will be triggered. However, the directional sector classification of AMD cannot accurately recognize the irregular road topology. Consequently, it fails to detect some transitions so it misses some opportunities to forward data messages.

## 3. Traffic Adaptive Data Dissemination

**Figure 1:**
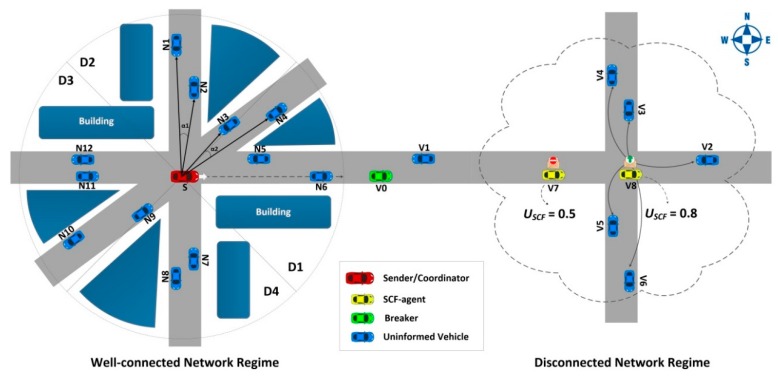
Illustration of the TrAD protocol.

The TrAD protocol is based on two main components. The first component adopts the broadcast suppression technique for well-connected networks, and the second one adopts a store-carry-forward mechanism for disconnected networks. Figure 1 presents the two running modes of the TrAD protocol according to the network scenarios: connected or disconnected network.

For a better understanding of this protocol, we define several concepts for TrAD. These concepts will be used throughout the paper:

- *Directional Cluster* $C_{d}$*:* The directional cluster is a group of vehicles in the one-hop neighborhood of a sender *S.* Moreover, these vehicles are travelling in a similar direction with respect to the sender *S*. In an ideal situation, *S* can accurately classify its neighbors into different directional clusters that match the different road topologies around it. For instance, *S* classifies its neighbors N1,  N2 into $C_{1}$ and N3,  N4 into $C_{2}$ as illustrated in Figure 1. The two directional clusters are exactly on the roads in two directions.
- *Coordinator:* This concept was firstly defined in the Greedy Perimeter Coordinator Routing (GPCR) protocol [24]. The *coordinator* is the vehicle located at an intersection (e.g., the transmitter *S* in the Figure 1).
- *Breaker:* In a well-connected network, the *breaker* is not only the furthest vehicle in the forwarding direction but also moves towards the outside of the vehicular network (e.g., the green vehicle V0 in the Figure 1).
- *SCF-agent:* The role of a SCF-agent vehicle is to be responsible for storing and carrying the data messages until it meets opportunities to forward (broadcast) the stored data messages to uninformed vehicles that come from other VANETs.
- *Beacon and Beaconing:* A beacon is defined as a periodic message that is responsible for exchanging the up-to-date status of nodes in the one-hop neighborhood. This periodic exchanging process is called beaconing. The beacon format is defined as following: < *Beacon ID*, *Sender ID*, *Global GPS Position*, *Heading*, *Number of Neighbors*, *Channel Busy Ratio*, *Message List* >.

**Figure 2:**
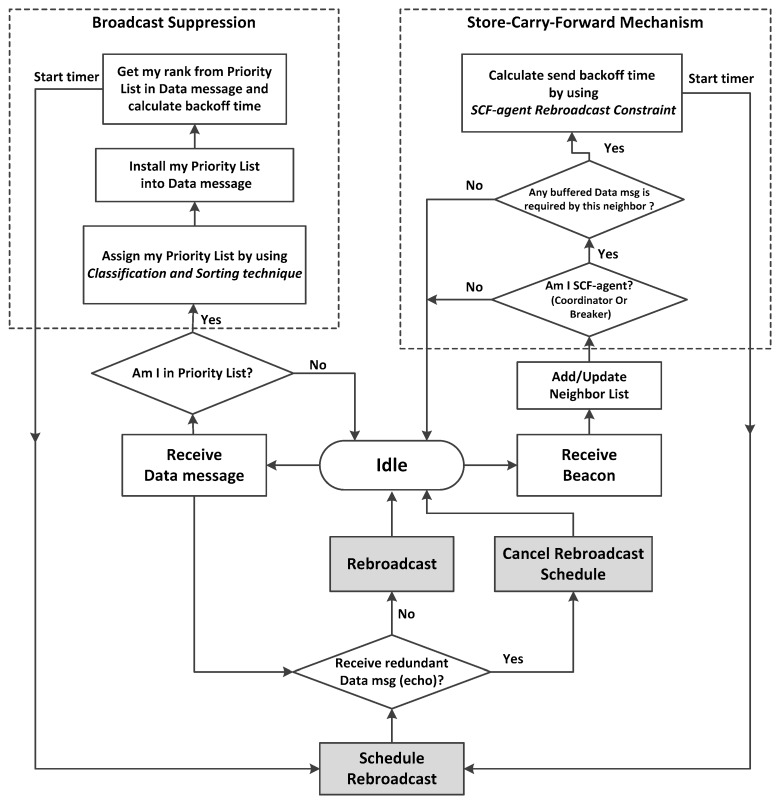
The operation of TrAD protocol flowchart.

In this section, the principle of operation for the TrAD protocol is presented as shown in Figure 2. TrAD requires that every vehicle send beacons to exchange their up-to-date status in the one-hop neighborhood. The period of beaconing $P_{b}$ is set to 1 s and the tolerance of beacon equals to $1.5\;P_{b}$ for a rapid reaction to the change of neighbors. The beaconing is another critical research field for safety applications, which includes several congestion control mechanisms, such as transmit power control, transmission rate control and encoding [25,26,27,28]. However, the adaptive beaconing is beyond the scope of this paper, we focus on the multi-directional and multi-hop data dissemination protocol. Therefore, we set a fixed beaconing interval for TrAD.

### 3.1. Broadcast Suppression Technique

To mitigate the broadcast storm in well-connected networks, first, a sender uses a classification mechanism to classify its neighbors into different directional clusters. Then, a sorting technique considers both road traffic and network traffic status to allocate different rebroadcast orders to the neighbors in directional clusters. After that, the transmitter inserts the rebroadcast order list, *i.e.*, priority list, into the data message and sends it. When the neighbors of the sender receive the data, a *delay-contention scheme* is used to convert the rebroadcast order to the respective delay and perform a rebroadcast schedule. Thus, the neighbors start to contest the opportunity to rebroadcast. A neighbor having higher order will rebroadcast earlier. If a vehicle receives redundant data (an echo) from a member of the same directional cluster, it will cancel its rebroadcast schedule and switch to an idle state. When the delay timer has expired, if a vehicle does not receive any data or just receives the data from the vehicles of other directional cluster, it will rebroadcast first. Therefore, the suppression technique not only can suppress redundant broadcasts, but also let the data messages be disseminated in all potential directions. Notice that the protocol makes a local centralized decision in the sender to decide the rebroadcast order of neighbors. In fact, the local centralized decision can solve the hidden terminal problem in the multi-directional dissemination [10,29].

#### 3.1.1. Vector-Angle-Based Cluster Classification Mechanism

A lightweight and flexible classification algorithm is designed, which only utilizes the position information rather than the digital road map. Therefore, it requires less computational power and fits more complex road topologies. Vector angle is used to identify whether the vehicles belong to a directional cluster or not. Algorithm 1 describes the steps used to classify directional clusters.

**Algorithm 1.** Vector-angle-based cluster classification algorithm**Condition:** the sender S receives a data message**Input :** the position of the sender S and its neighbors N(S)
1:
**while**
N(S)≠∅
**do**2:
**for** i = 1 to num[N(S)], d = 1 **do**3:
$N\left(S\right)=N\left(S\right)-\left\{n_{i}\right\}⇐$ eliminate $n_{i}$ from N(S)
4:
$C_{d}\leftarrow n_{i}⇐$ sort $n_{i}$ into directional cluster $C_{d}$
5:
**for** j = i to num [N(S)] **do**6:
**if**$\;\hat{(n_{i}Sn_{j}}<\alpha$) **then**7:
$C_{d}\leftarrow n_{j}⇐$ sort $n_{j}$ into the same $C_{d}$ of $n_{i}$
8:
$N\left(S\right)=N\left(S\right)-\left\{n_{j}\right\}$
9:
**end if**10: j++11:
**end for**12: save
$C_{d}$ into cluster buffer13: d++14:
**end for**15:
**end while**

The cluster classification operation is conducted as follows: In the well-connected network regime of Figure 1, the sender or transmitter *S* extracts the first neighbor N1 from its neighbor list and classifies N1 into the directional cluster $C_{1}$.

Knowing the *N1* and *N2* coordinates, the angle $\alpha_{1}$ between $\vec{SN1}$ and $\vec{SN2}$ can be obtained from their dot product ($\alpha_{1}\in \left[0,\;\pi \right]$). The algorithm compares $\alpha_{1}$ with a threshold angle α, e.g., α=10°. If the angle $\alpha_{1}$ is less than α, the neighbor N2 is classified in the directional cluster $C_{1}$. If not, the neighbor N2 still remains in the neighbor list for the next step. In the case of Figure 1, $\alpha_{1}<\alpha$, so $N2\in C_{1}$. This process continues until all neighbors have been checked. Therefore, a group of vehicles $C_{1}$ that are located in the similar direction as N1 is identified. The members of $C_{1}$ are saved in a cluster buffer and eliminated from the next classification step. The classification will be carried out in a continuous process until all neighbors have been classified into respective directional clusters.

The classification result of TrAD in the case of well-connected network illustrated by the Figure 1 is C_1_ = {*N1*, *N2*}; C_2_ = {*N3*, *N4*}; C_3_ = {*N5*, *N6*}; C_4_ = {*N7*, *N8*}; C_5_ = {*N9*, *N10*}; C_6_ = {*N11*, *N12*}. Vehicles located in the similar direction with respect to *S* are exactly classified in the same directional cluster. Moreover, the AMD classification result is also illustrated in Figure 1. AMD divides the radio range into four equal sectors, *i.e.*, D1, D2, D3 and D4. Notice that the AMD classification result is not accurate. The C_2_= {*N3*, *N4*} and C_3_= {*N5*, *N6*} are combined into sector D1, and the C_5_ = {*N9*, *N10*} and C_6_ = {*N11*, *N12*} are mixed into sector D3. Furthermore, the vehicle *N3* and *N9* are ambiguous on the boundary of sectors. The result proves that the directional sector classification of AMD is not appropriate for the irregular road topology.

#### 3.1.2. Traffic Adaptive Sorting Technique

The algorithm takes into account both road traffic and network traffic status. Three metrics are considered: the number of neighbors N, the distance between a sender and its neighbor D, and the Channel Busy Ratio CBR. The metrics N and D represent the road traffic condition, and CBR reflects the network traffic status. The sender *S* uses these three metrics to estimate the utility $U_{TX}$ and detects the channel congestion for every neighbor. First of all, the metrics must be quantified for the calculation of utility $U_{TX}$. The value of all metrics falls in [0, 1].

The metric N (Equation (1)) indicates the data coverage of potential vehicles in the next transmission:

(1) $$N=\text{min}\left(\frac{\#Neighbor}{max.\#Neighbor}\;,\;1\right)$$

#Neighbor is the instantaneous number of neighbors in the sender vicinity. For the simulation, max.#Neighbor is a preset maximum number of neighbors, and it is set to 25. The metric D (Equation (2)) represents the distance between a sender and its neighbor. $Dist_{sn}$ is the distance measured between the sender and its neighbor. max.RadioRange is the maximum communication range of the wireless access medium.

(2) $$D=\min\left(\frac{Dist_{sn}}{max.RadioRange}\;,\;1\right)$$

The metric CBR (Equation (3)) describes the bandwidth occupation in a detection time interval $T_{interval}$, e.g., 1 s. The channel busy time $T_{busy}$ is detected by the Clear Channel Assessment (CCA) in the PHY layer of IEEE 802.11p:

(3) $$CBR=\frac{T_{busy}}{T_{interval}\;}$$

Therefore, we propose an equation (Equation (4)) to assign a higher transmission utility $U_{\text{TX}}$ to the neighbor that has more neighbors, further distance and free channel load. N and D are of equal importance to represent road traffic status. Finally, the $U_{TX}$ is scaled based on a weight $\omega_{CBR}$ that is related to network traffic status, *i.e.*, CBR:

(4) $$U_{TX}=\;\omega_{CBR}\cdot \left(\frac{N\;+\;D}{2}\right)$$

**Figure 3:**
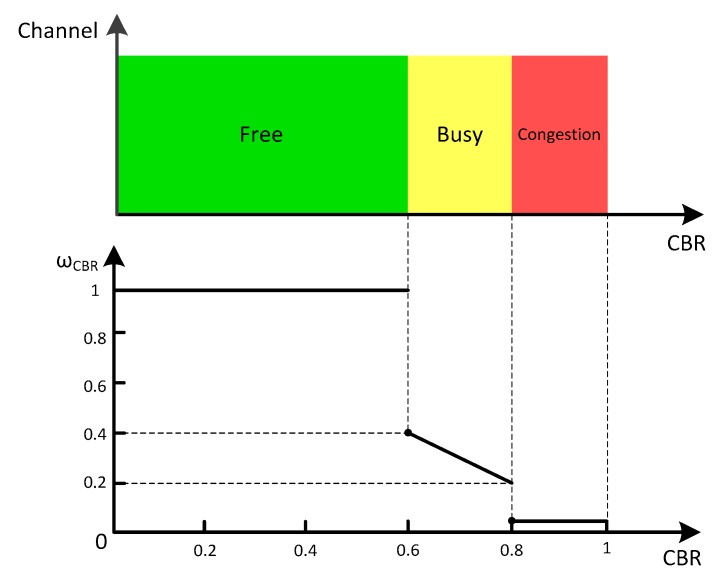
The projection of CBR on the weight $\omega_{\text{CBR}}$ and channel status.

$\omega_{CBR}$ is defined as a piecewise linear function (Equation (5)) in terms of CBR. The mapping of CBR on the $\omega_{CBR}$ and the channel status is shown in Figure 3. The network load is divided into three levels according to CBR, *i.e.*, free, busy and congestion:

(5) $$\omega_{CBR}=\{\begin{array}{cc} \;1 & \;0<CBR<0.6\\ 1-CBR & \;0.6\leq CBR<0.8\\ \;0.001 & \;0.8\leq CBR<1 \end{array}$$

Some well-known studies agreed that the suitable CBR target value for safety applications is 0.6 [30,31]. Fallah *et al.* identified an appropriate maximum CBR as 0.8 [32]. Therefore, $\omega_{CBR}$ does not affect $U_{TX}$ until CBR achieves 0.6. Then, $\omega_{CBR}$ decreases with the increasing of CBR, when CBR is between 0.6 and 0.8. If CBR is more than 0.8, a very small value, *i.e.*, 0.001, will be assigned to $\omega_{CBR}$ to minimize $U_{TX}$. Thus, the sender *S* will sense the congestion of this neighbor and a lowest rebroadcast order is allocated to the neighbor to mitigate the congestion. Moreover, if a sender detected that all the members of a cluster are congested, the cluster will be deleted from the priority list. If the network load of the cluster is alleviated to free or busy level later, the transmission will be recovered.

**Figure 4:**
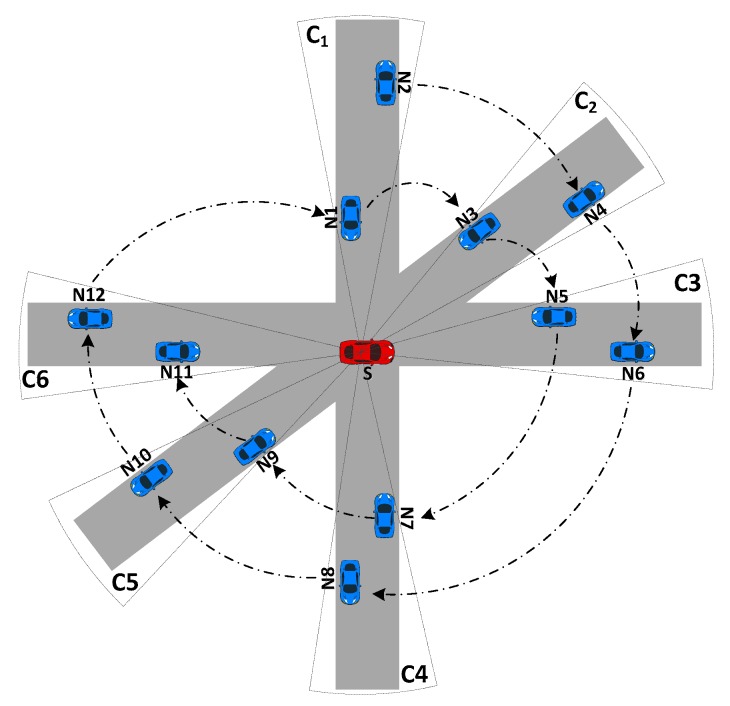
The sorting process for a priority list.

As stated in Equation (4), the neighbors in every directional cluster are sorted in decreasing order in terms of their utility. Then, a round-robin fashion is used to define the final order of vehicles in the priority list [10]. For clear explanation, Figure 4 is given to illustrate the process of the sorting technique. The vehicle having the highest $U_{TX}$ in the directional cluster C_1_ is put into the first position of the priority list, followed by the vehicle having highest $U_{TX}$ in directional cluster C_2_, *etc*. This method guarantees the fairness for each directional cluster. For instance, we assume that the utility $U_{TX}$ of vehicles is proportional to its ID number in Figure 4, e.g., $U_{N2}>U_{N1}$, so after the first round of sorting, the priority list is {*N2*, *N4*, *N6*, *N8*, *N10*, *N12*}. In the second round, all neighbors are sorted and the final priority list is {*N2*, *N4*, *N6*, *N8*, *N10*, *N12*, *N1*, *N3*, *N5*, *N7*, *N9*, *N11*}.

The sender S inserts the priority list into the header of the data message before transmitting. The header format is < *Data ID*, *Originator ID*, *Sender ID*, *Originator Position*, *Sender Position*, *Priority List* >. When the recipients receive a data message from *S*, they extract the priority list and seek out their rank R ∈[0, n−1], where n is the number of neighbors. TrAD gives one time interval ti between the former transmission and the latter one. ti includes the total time taken from the transmitter to the recipient, such as medium access delay, propagation delay and transmission delay. The delay (back-off time) of transmissions $Delay_{TX}$ is obtained by using Equation (6):

(6) $$Delay_{TX}=ti\cdot R$$

### 3.2. Store-Carry-Forward Mechanism

TrAD assigns the role of SCF-agent to the vehicles at a specific location in a VANET, which is beneficial to fill the connectivity gaps among vehicular clusters. The SCF-agent stores and carries data messages from one cluster to another, and then it will forward (broadcast) the stored data to the new coming vehicles. That is, TrAD uses the temporal delay to complement the spatial partition. Moreover, we designed a SCF-agent rebroadcast constraint technique that is a particular operation to *further reduce* the redundant broadcasts. In order to avoid data redundancy, the protocol compares the message list of a SCF-agent with its neighbors’. Only the data messages required by the neighbor will be scheduled by using the SCF-agent rebroadcast constraint technique.

#### 3.2.1. The Selection of SCF-Agent

To facilitate the understanding, we introduce some techniques which enable TrAD to identify the coordinator and the breaker.

**Figure 5:**
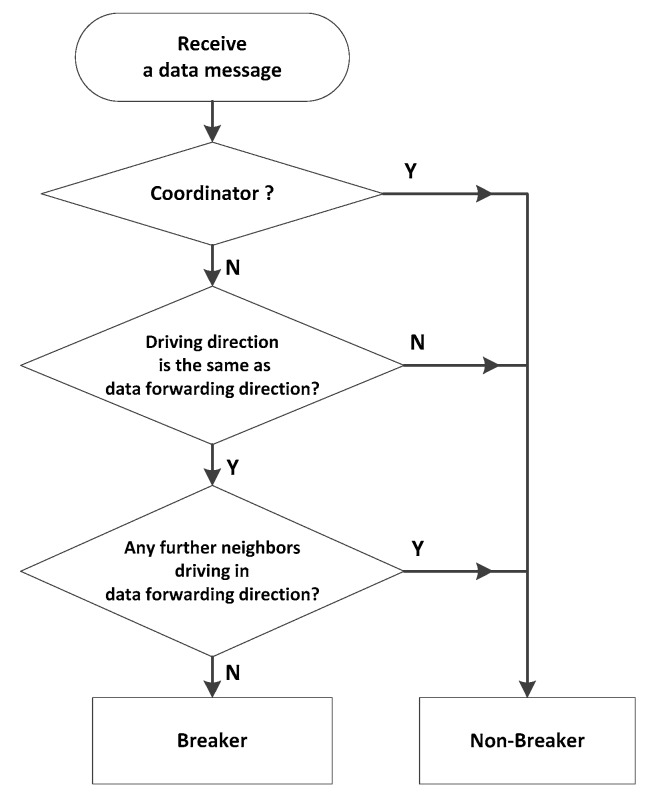
The identification of a breaker flowchart.

- *Coordinator:* After preloading an intersection position list of the urban map into the system; every vehicle will detect the distance between itself and all intersections when the vehicle receives a beacon message. If the distance is less than 20 m, the vehicle is defined as a coordinator. *Breaker*: The flow chart of identification of a breaker is shown in Figure 5. When a vehicle receives a data message, it will detect and eliminate the possibility of the coordinator role. Then, the vehicle determines whether its moving direction is the same as the data forwarding direction. If so, the vehicle will look up whether there is a further neighbor moving at the front. If not, the vehicle is defined as the breaker. In particular, this procedure is carried out at a continuous process until the boundary of the well-connected network is established.

Vehicles that satisfy any of the definitions of coordinator and breaker are selected as SCF-agents. This operation is compatible with both urban and highway scenarios. The coordinator is specialized for the two-dimensional topology of urban scenarios. At the intersection, a coordinator obviously has more chances to connect to neighbors than the vehicles located between intersections. Therefore, TrAD chooses coordinators to store and carry the data, which is beneficial to discover more uninformed vehicles. Given that there is no coordinator in highway scenarios, the breaker is defined for both urban and highway scenarios. It can move towards outside of the well-connected network and transmit the data message into a wider area.

#### 3.2.2. SCF-Agent Rebroadcast Constraint Technique

The rebroadcast constraint technique aims to avoid redundant rebroadcasts when multiple SCF-agents receive an identical requirement (beacon) simultaneously. If the protocol does not have such a constraint technique, these SCF-agents would transmit the same data messages at the same time, and redundant broadcasts and collisions will be incurred. Thus, we also use the delay-contention scheme to avoid the redundancy. This method can calculate different delays (back-off times) according to the utility $U_{SCF}$ of every SCF-agent. If a SCF-agent receives an echo message (redundant data message) that is the same as the one it is scheduling, the SCF-agent will cancel the forwarding schedule and switch to an idle state. If not, the SCF-agent will transmit first when the timer expires.

Contrary to the utility $U_{TX}$ in broadcast suppression technique, here, a SCF-agent is given a higher utility $U_{SCF}$ when the SCF-agent and an uninformed vehicle are closer. Indeed, a shorter distance is helpful to guarantee a better QoS of consecutive transmissions. Furthermore, since the number of neighbors neither contributes to SCF transmission nor constrains the redundant rebroadcast, $U_{SCF}$ does not consider it. The weight $\omega_{CBR}$, representing network status, is also used to scale $U_{SCF}$ (Equation (7)):

(7) $$U_{SCF}=\omega_{CBR}\cdot \left(1-D\right)$$

It is to be noted that, in general, a new incoming (uninformed) vehicle does not require only a single data message, but several. Especially at the boundary of ROI, the vehicle going out of the ROI will perform consecutive transmissions to the new vehicle incoming into the ROI. If the protocol does not handle this scenario properly, a burst of transmissions will be incurred. Therefore, TrAD uses Equation (8) to calculate transmission delay $Delay_{SCF}$ to solve this problem:

(8) $$Delay_{SCF}=\;ti\cdot \left[BurstCount+\left(1-U_{SCF}\right)\right]$$

One time interval ti is set between each transmission of required data messages. The BurstCount is the transmission order of data messages. A SCF-agent with a higher utility $U_{SCF}$ will fire sooner to transmit the data messages in its transmission queue. The $ti\cdot \left(1-U_{SCF}\right)$ part also performs as an additional delay, by which the time slot boundary synchronization problem can be mitigated.

For example, in the disconnected network illustrated by the Figure 1, vehicles V7 and V8 are SCF-agents moving from the well-connected network and carrying the same data messages. They received simultaneously the requirement (beacon) from their neighbor vehicles. Since the utility $U_{SCF}$ of V8 (0.8) is greater than that of V7 (0.5), V8 is triggered to broadcast the data messages first. Consequently, V7 hears the redundant data message, and then cancels the respective schedule. Therefore, the redundant transmissions of V7 are suppressed.

## 4. Performance Evaluation

To evaluate the performance of the TrAD protocol, we make an important effort to implement realistic simulations in both urban and highway scenarios. Each simulation *scenario* has a map and traffic routes. The *map* is composed of the road topology and the building obstacles. The *traffic route* defines the planned round-trip route for each vehicle cluster. Notice that several traffic routes comprise the road traffic network on the map. The departure of vehicles follows an exponential distribution law. To perform an objective performance evaluation, the simulation programs are implemented by including three state-of-the-art IVC protocols: Distributed Vehicular Broadcast (DV-CAST) for highway scenarios [8], Urban Vehicular Broadcast (UV-CAST) for urban scenarios [9] and Adaptive Multi-directional data Dissemination (AMD) for both urban and highway scenarios [10].

- *DV-CAST* is a distributed broadcast protocol for highway scenarios with zero infrastructure support. The protocol resorts to neighbor detection to distinguish between vehicles *Vs* driving in the same direction with respect to the source vehicle and those *Vo* driving in the opposite direction. If the vehicles *Vs* are connected to each other, the broadcast suppression technique will be used in multi-hop broadcasts. However, if there are gaps between clusters, the store-carry-forward mechanism will be performed to select some of vehicles *Vo* to be SCF-agents. In the simulation of this paper, we use the slotted 1-persistence broadcast suppression technique and set the total number of time slots $N_{st}$ to 5, the slot time st to 5 ms.
- *UV-CAST* is a distributed broadcast protocol specialized for infrastructure-less urban VANETs. The protocol uses a gift-wrapping algorithm to select boundary vehicles to store, carry and forward messages to fill the gap between different partitioned or disconnected networks. It also uses a lightweight suppression technique for well-connected networks. This technique assigns a higher priority to the vehicle located at an intersection to rebroadcast, which can disseminate the data message in more directions and suppress the redundant broadcast. In the simulation of this paper, we set the maximum waiting time $\tau_{\text{max}}$ to 500 ms that is the same as the simulation setting in the original paper. Furthermore, a position list of intersections is preloaded in the system. If the distance between a vehicle and one of intersections is less than 20 m, the vehicle is believed to be at the intersection.
- *AMD* is an infrastructure-less data dissemination protocol supporting both highway and urban scenarios. A generalized time slot scheme is used to suppress broadcasting, which is based on directional sectors. The number of directional sector is adjusted adaptively between two or four according to the local road topology and the distribution of neighbors. A store-carry-forward mechanism resorts to a role transition, *i.e.*, from *tail* to *non-tail*, to trigger a new broadcast to uninformed vehicles. In the simulation of this paper, the time slot st is set to 5 ms and one time slot is assigned to one vehicle, *i.e.*, $ts_{d}=1$. Moreover, the additional delay ${\text{AD}}_{\text{ij}}$ is set to one DIFS, *i.e.*, 58 μs. The position list of intersections is preloaded in the system and is operated as the UV-CAST case.

Veins 3.0 framework is used as the basic library to implement the entire wireless communication protocol stack. It is to be noted that the Veins framework is based on two simulators: OMNeT++ 4.4.1, and SUMO 0.23.0. OMNeT++ is an event based network simulator, and SUMO 0.23.0 is a road traffic simulator. The MAC and PHY layer of the DSRC device is based on the IEEE 802.11p standard. The data rate is set to the maximum data rate for broadcasting (6 Mbit/s in IEEE 802.11p), the transmission power is set to 300 mW (the legal power in US is less than 1000 mW). The Friis Free Space Path Loss (FSPL) propagation model is used, in which the exponent α of FSPL is assigned to 3.0, for the value from 2.7 to 5.0 is estimated for the outdoor shadowed urban environment [33]. According to the setting of propagation model, the radio communication range is about 366 m. For the Bit Error Rate (BER) model of 6 Mbit/s data rate proposed by K. Sjöberg, *et al.* is implemented [34]. We adopt the empirical model of IEEE 802.11p radio shadowing in urban environments proposed by Sommer *et al.* [35]. For all simulations, the size of data messages is set to the maximum single packet size allowed by the IEEE 802.11p standard (*i.e.*, 2312 bytes), because we would like simulate the worst case of the network traffic. To be noted that each source node broadcasts periodically the data message every 2 s. The broadcast frequency could be changed to adapt to the requirements of an application. In this work, we use the same broadcast frequency for evaluating all the investigated protocols in the defined scenarios. Moreover, the size of beacon is set to 378 bytes, where the entry number of message list is set to 40. The beacon sending period is 1 s, and we focus mainly on the simulation of one-hop neighborhood worst case refresh update. The vehicular densities are from 40 v/km^2^ to 160 v/km^2^, the interval is 20 v/km^2^. The simulation setting parameters are summarized in Table 1.

The quantitative analysis considers the following metrics:

- *Packet Delivery Ratio (PDR):* It represents the average number of data messages received by the vehicles in the ROI as a percentage of the total number of data messages sent by the source node. It also can be called the coverage of data dissemination.
- *Number of Transmissions:* It is the total number of transmissions performed by the vehicles for broadcasting in the ROI during the whole simulation time.
- *Delay:* It is the average time interval between the sending of a data message by the source and its reception by the vehicles in the ROI.
- *Data Dissemination Speed:* This metric represents how fast the data can be disseminated among vehicles in the ROI. To record this metric, the source only sends data message once, and then the simulation computes the increase of data coverage with time. If the data coverage increases greatly in a short time, which means the protocol achieves a high dissemination performance. This metric is mainly used to estimate the impact of GPS drift on the performance of protocols.

To comprehensively evaluate the performance of TrAD, various experiments were designed to analyze the impact of different urban maps, different traffic routes, network density and GPS drift. The geographic data were retrieved from OpenStreetMap (OSM) database. Traffic rules, e.g., the speed limitation and the traffic light, were set according to the real world scenarios. By using the SUMO tool chain, all the geographic and traffic data were converted to the SUMO file format.

### 4.1. Different Urban Maps

**Figure 6:**
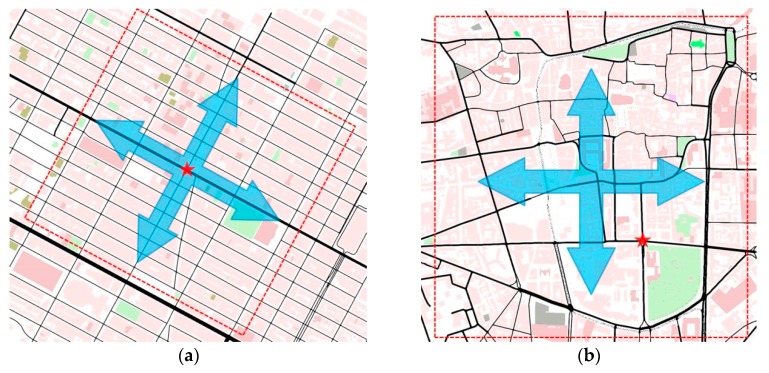
Urban Maps: (**a**) Manhattan Island in New York City (USA); (**b**) Clermont-Ferrand (France); Traffic routes are indicated by arrows in urban scenarios; The red start is the source node that periodically broadcasts data messages; The red broken line indicates the boundary of ROI.

For urban scenarios, two maps were created. The first map is a fragment of Manhattan Island in New York City, USA (Figure 6a), and the second map is the downtown area of the city of Clermont-Ferrand, France (Figure 6b). The ROI size of both two maps is 1 × 1 km^2^. The map statistics, as shown in Table 2, indicate that the map of Clermont-Ferrand has more lanes, junctions and shorter street lengths than the Manhattan one, which means that the road traffic topology is more complex in Clermont-Ferrand than in Manhattan.

**Figure 7:**
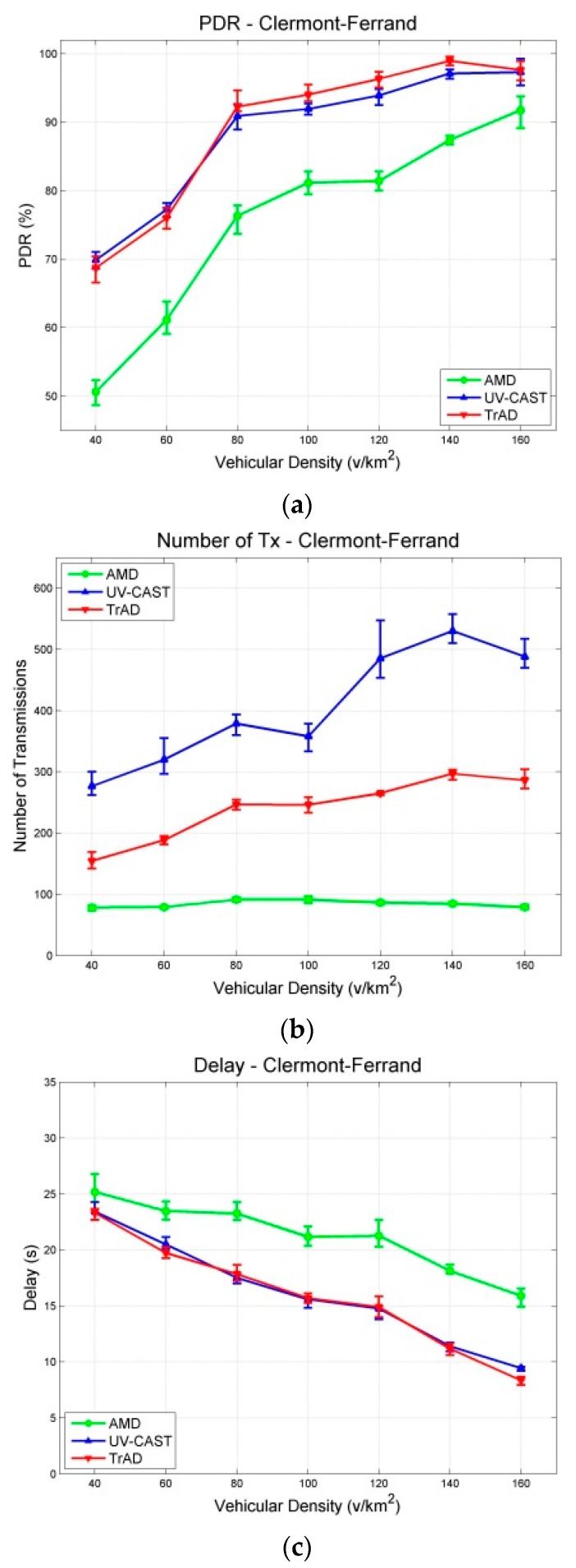
Results with a 95% confidence interval in Clermont-Ferrand scenarios: (**a**) PDR; (**b**) Number of Transmissions; (**c**) Delay.

**Figure 8:**
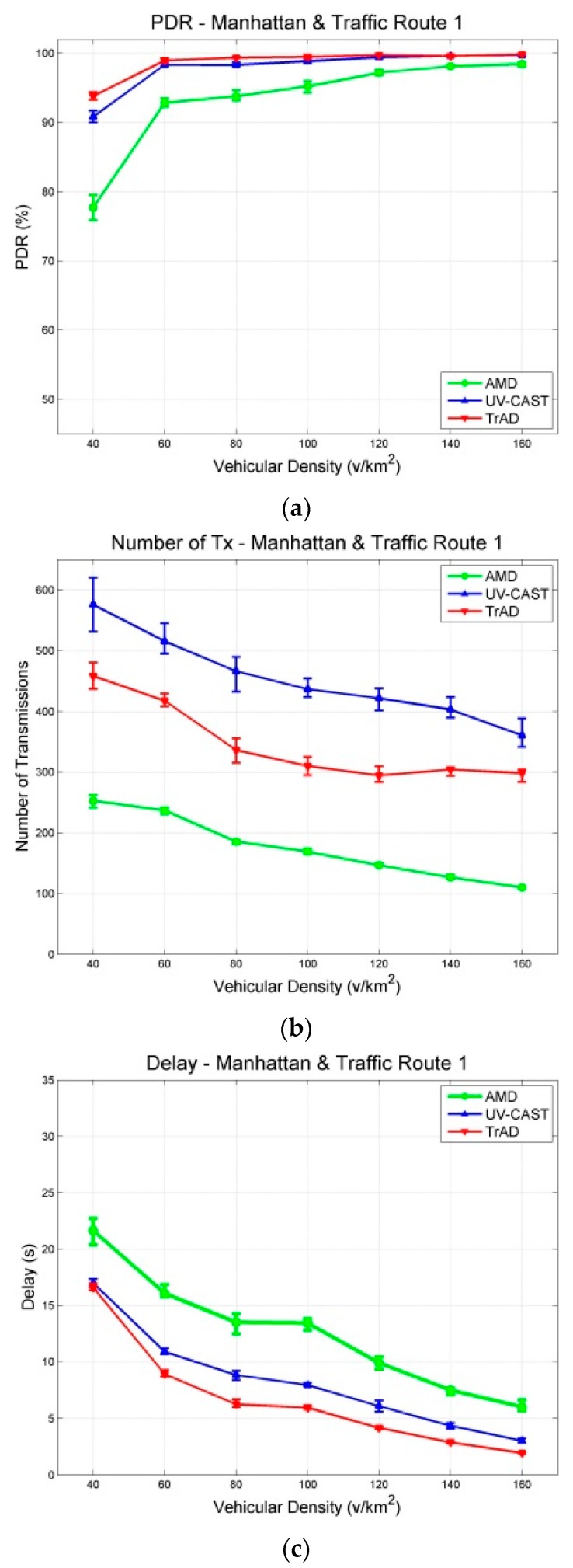
Results with a 95% confidence interval in Manhattan scenarios with the traffic route 1 (uniform): (**a**) PDR; (**b**) Number of Transmissions; (**c**) Delay.

The data messages are generated by a fixed source node located at an appropriate intersection and are collected by all vehicles moving in the ROI. Similar traffic routes were created for two maps, where vehicles are distributed uniformly in the scenarios. The experiment of each vehicular density is performed for 400 s and repeated 10 times with different random seeds. The results with a 95% confidence interval are shown in Figure 7 and Figure 8. The junction points of the curve are the average values of the metric in repeated simulations. The upper and lower error bar represents the maximum and the minimum, respectively.

As expected, the obtained simulation results show that the different urban maps significantly impact the wireless communication protocols because the scenarios affect the network topology and network link quality, respectively. According to the simulation results of the two scenarios, the Packet Delivery Ratio (PDR) of AMD is the worst in both the Clermont-Ferrand and Manhattan scenarios. Therefore, we compare firstly the performance of TrAD and UV-CAST on different maps.

Figure 7a shows that the PDR of TrAD and UV-CAST increases rapidly from about 70% to over 90% when the vehicular density increases from 40 v/km^2^ to 80 v/km^2^, and then continues to gradually reach close to 100%. In case of the Manhattan map (Figure 8a), the PDR of TrAD and UV-CAST already achieves over 90% when the vehicular density equals 40 v/km^2^, and then reaches close to 100% when the vehicular density is 80 v/km^2^. Notice that the PDR of TrAD is better than the UV-CAST one (93.82% *vs.* 90.84%) at the starting point of vehicular densities (*i.e.*, 40 v/km^2^). The reason for these observations lies in the different complexity of the two maps. The Manhattan map, a regular one, is relatively easy to handle with the protocols. On the contrary, the complex road traffic environment of the Clermont-Ferrand map, *i.e.*, winding and short streets, T style intersections and irregular buildings, leads to intermittent connectivity and increases the complexity of the road topology recognition.

In fact, UV-CAST does not consider the road topology to classify vehicles into different directional clusters. It assigns a high priority to the vehicles located at an intersection to rebroadcast the data messages and use a gift-wrapping algorithm to select the boundary vehicles to be the SCF-agent. As the authors of UV-CAST admitted, the gift-wrapping algorithm would over-select the SCF-agent vehicles. On one hand, it seems that UV-CAST cannot control appropriately the number of transmissions. Thus, the number of transmissions of UV-CAST is much higher than the one of TrAD (Figure 7b and Figure 8b). On the other hand, TrAD uses the vector-angle-based classification mechanism to sort vehicles into respective directional cluster that matches the road topology. Moreover, TrAD also resorts to the traffic adaptive sorting technique to fairly classify vehicles into the priority list. Therefore, the redundant data message transmissions are canceled appropriately in each directional cluster and, at the same time, the data messages are disseminated more efficiently into every possible direction in the ROI.

Figure 7c and Figure 8c indicate that the delay of TrAD and UV-CAST in both the Clermont-Ferrand and Manhattan scenarios decreases with increasing vehicular density. Moreover, the delay in the Clermont-Ferrand scenario is higher than the one in the Manhattan scenario for the respective vehicular densities. This shows the side effect of the complex road topology of Clermont-Ferrand scenarios. We also observe that TrAD achieves a similar level of delay as UV-CAST in the Clermont-Ferrand scenarios and gains an advantage over UV-CAST in the Manhattan scenarios. The negative impact of complex scenarios also influences the PDR of protocols, it narrows the difference of PDR between TrAD and UV-CAST, but UV-CAST consumes more transmissions.

For AMD and TrAD, in Figure 7a and Figure 8a, the first observation attracting our attention is that the difference of PDRs between AMD and TrAD is greatly narrowed from the Clermont-Ferrand scenarios to the Manhattan cases. That is, AMD fits much better a regular map (e.g., Manhattan) than a complex one (e.g., Clermont-Ferrand). Even so, the PDR and the delay of AMD are still worse than the TrAD ones. However, AMD is suitable to be used as reference, since, like TrAD, it also adopts a directional classification mechanism in the broadcast suppression technique. The difference lies in that TrAD can provide a more precise classification than AMD to handle more complex road topologies. This advantage is proved by the fact that the PDR of TrAD is always better than the one of AMD in both scenarios. Although AMD consumes the least transmissions, TrAD uses a moderate amount of transmissions (a little bit more network overhead) to achieve a better overall performance.

### 4.2. Different Traffic Routes

For urban scenarios, most of previous work used different maps to evaluate the performance of IVC protocols. Notice that the different traffic routes on a same map may impact significantly the performance of IVC protocols. Therefore, we will not only analyze the impact of different maps, but also investigate the impact of different traffic routes. The simulation of each vehicular density is performed for 400 s and repeated 10 times with different random seeds.

**Figure 9:**
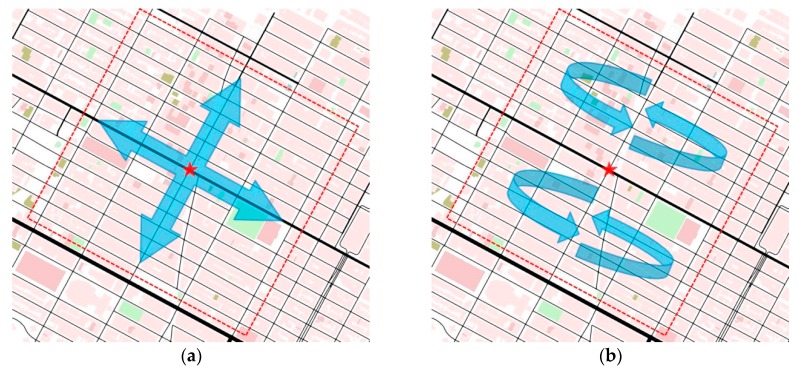
Traffic routes in the Manhattan scenario. (**a**) Traffic route 1 (uniform); (**b**) Traffic route 2 (deviated); Traffic routes are indicated by arrows in urban scenarios; The red start is the source node that periodically broadcasts data messages; The red broken line indicates the boundary of ROI.

**Figure 10:**
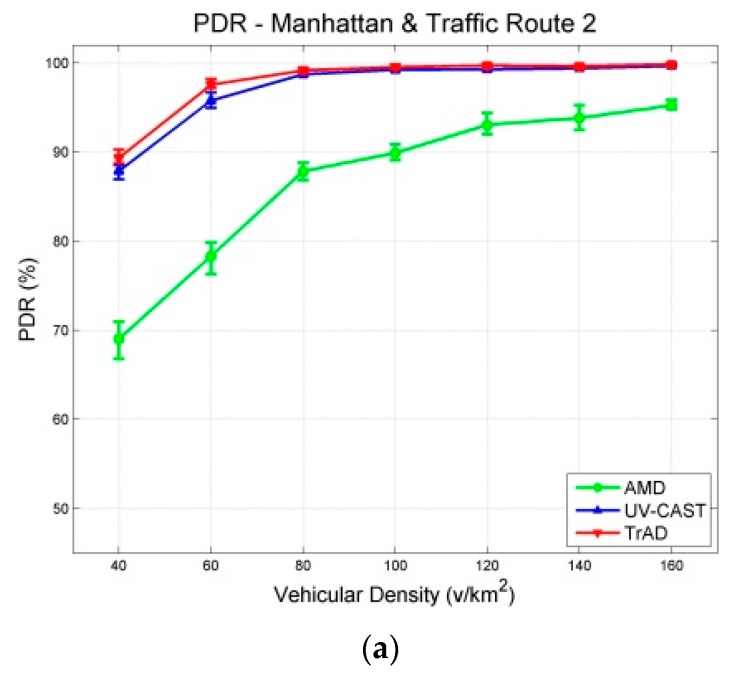

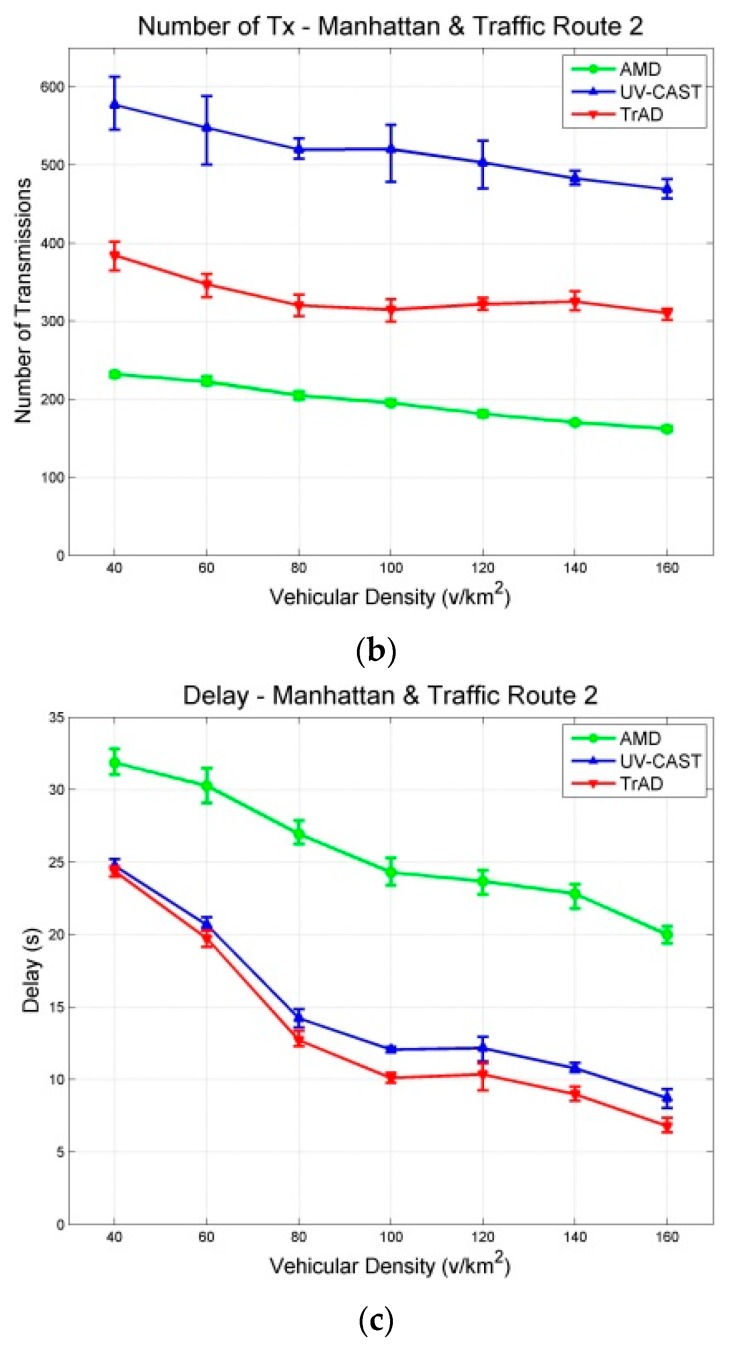
Results with a 95% confidence interval in Manhattan scenarios with the traffic route 2 (deviated): (**a**) PDR; (**b**) Number of Transmissions; (**c**) Delay.

The source node is fixed at the center of the Manhattan map and two different traffic routes that represent two types of road traffic network are designed, as shown in Figure 9. The arrows indicate traffic routes: Traffic route 1 (Figure 9a) includes vehicles driving between the top and the bottom half of the scenario, so that they can cross the middle line of the map where the source node is located. Therefore, the vehicles get more chance to connect to the source node or other vehicles. On the contrary, traffic route 2 (Figure 9b) constrains vehicles inside the top or the bottom half of the scenario. In this case, only a few vehicles pass by and connect to the source node. That means traffic route 2 creates more disconnected networks than the ones of traffic route 1. Therefore, the design of different traffic routes mainly aims at evaluating the performance of IVC protocols to handle the disconnected network problem. Notice that the scenario with traffic route 1 (Figure 9a) is the same as the Manhattan urban scenario (Figure 6a). Thus, the simulation results of these two experiments are also the same. The results of different traffic routes are shown in Figure 8 and Figure 10.

In Figure 8a and Figure 10a, it is obvious that the PDR of AMD is significantly affected by the different traffic routes. In scenarios with traffic route 1, the PDR of AMD starts from about 80% at the first vehicular density, *i.e.*, 40 v/km^2^, and exceeds 90% at the second vehicular density, *i.e.*, 60 v/km^2^, finally reaches close to 100%. In the case of traffic route 2, it starts from about 70%, and just achieves 90% at the vehicular density 100 v/km^2^, finally reaches about 95%. These observations reflect that the SCF mechanism of AMD cannot well handle the deviated traffic route or the severe disconnected network. On the other hand, TrAD and UV-CAST are more robust than AMD to the change of traffic routes. Their PDRs only decrease slightly at sparse densities, *i.e.*, 40 v/km^2^ and 60 v/km^2^, in scenarios with traffic route 2 compared with the ones with traffic route 1. Notice that TrAD gains a slight advantage of PDR over UV-CAST at sparse densities in both scenarios.

Although the PDR results of TrAD and UV-CAST are close, the number of transmissions of these two protocols is different. The number of transmissions of TrAD is always lower than the one of UV-CAST in scenarios with both traffic routes, as shown in Figure 8b and Figure 10b. The reason lies in that TrAD uses the suppression technique in the broadcast and the SCF mechanism to efficiently constrain transmissions. However, the SCF mechanism of UV-CAST selects too many SCF-agent vehicles so as to trigger more redundant transmissions. It is worth mentioning that the difference of the number of transmissions between TrAD and UV-CAST is enlarged in scenarios with traffic route 2 compared with the ones with traffic route 1. This shows that TrAD gains more advantage over UV-CAST in scenarios with the deviated traffic route. The lowest number of transmissions is achieved by AMD, but the low PDR and the high delay let this advantage completely fade.

Figure 8c and Figure 10c present that traffic route 2 lets the delay of all protocols increase compared with the one of traffic route 1. Especially, for AMD, its average delay of all vehicular densities increases 13.117 s. The different between AMD and TrAD increases more than twice. In addition, TrAD obtains the lowest delay in both uniform and deviated traffic routes. These observations show that TrAD can handle the deviated traffic route in the Manhattan scenario.

A deviated traffic route, *i.e.*, traffic route 2, indeed influences the performance of IVC protocols significantly. It requires IVC protocol to consume more transmissions to maintain data coverage with high delay. However, TrAD shows an outstanding capability to handle this issue. The experimental observations show that TrAD only consumes a moderate amount of transmissions to handle the disconnected networks to achieve high data coverage with low delay. The reason lies in that TrAD has an efficient SCF mechanism that assigns the role of SCF-agent to the breaker and the coordinator. Furthermore, TrAD also uses a SCF-agent rebroadcast constraint technique to further suppress transmissions. For UV-CAST, the gift-wrapping algorithm triggers more transmissions, which also brings the extra delay. For AMD, although it could recognize the relatively regular Manhattan map, there is a drawback in its SCF mechanism. The SCF mechanism only triggers the transmission of stored data while the transition, *i.e.*, from tail to non-tail, happens, but, due to signal fading or interference, the stored data is not always received, especially in the scenarios with deviated traffic routes. In case a transmission fails, there is not another chance to trigger the SCF-agent to forward the stored data again.

### 4.3. Network Density

#### 4.3.1. Urban Scenarios

The obtained simulation results of different maps and different traffic routes in urban scenarios have been analyzed and elaborated. We observe that the network density, the map and the traffic route are the three main parameters which impact significantly the performance of IVC protocol. In general, a lower network density leads to more disconnected networks, which decreases the overall performance of the IVC protocols: low PDR, high delay and high number of transmissions (network overhead). On the contrary, a higher network density enables the IVC protocols to achieve better overall performance. However, the geographic and traffic environment could also affect significantly the performance of IVC protocols, *i.e.*, the complex road topology, high-rises and the deviated distribution of vehicles. Therefore, to implement a high performance IVC protocol, it is important to consider the abovementioned elements.

#### 4.3.2. Highway Scenarios

**Figure 11:**
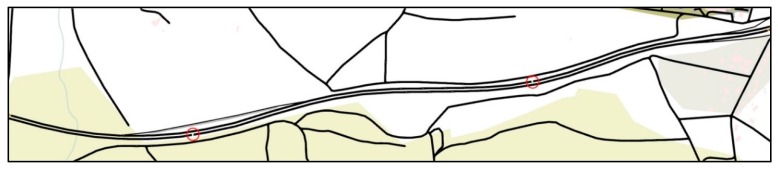
A711 highway of Clermont-Ferrand, France.

**Figure 12:**
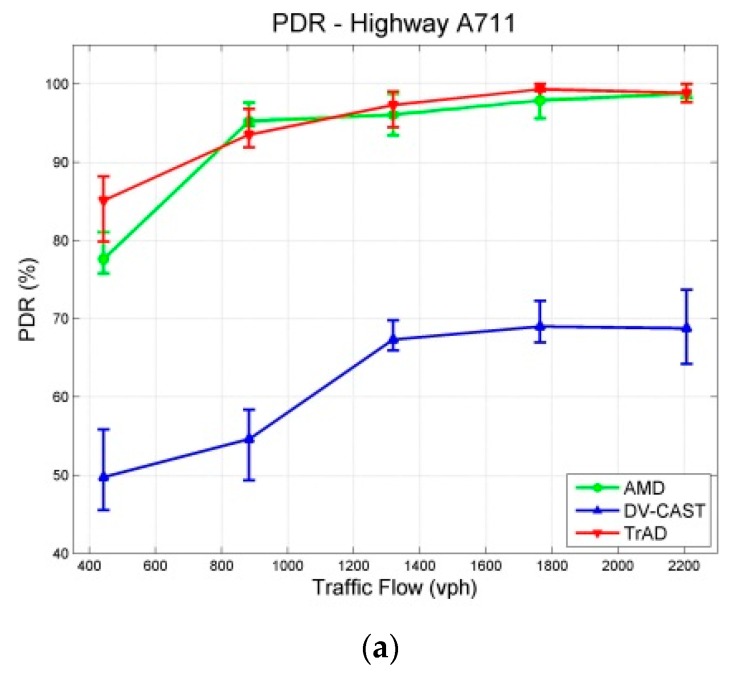

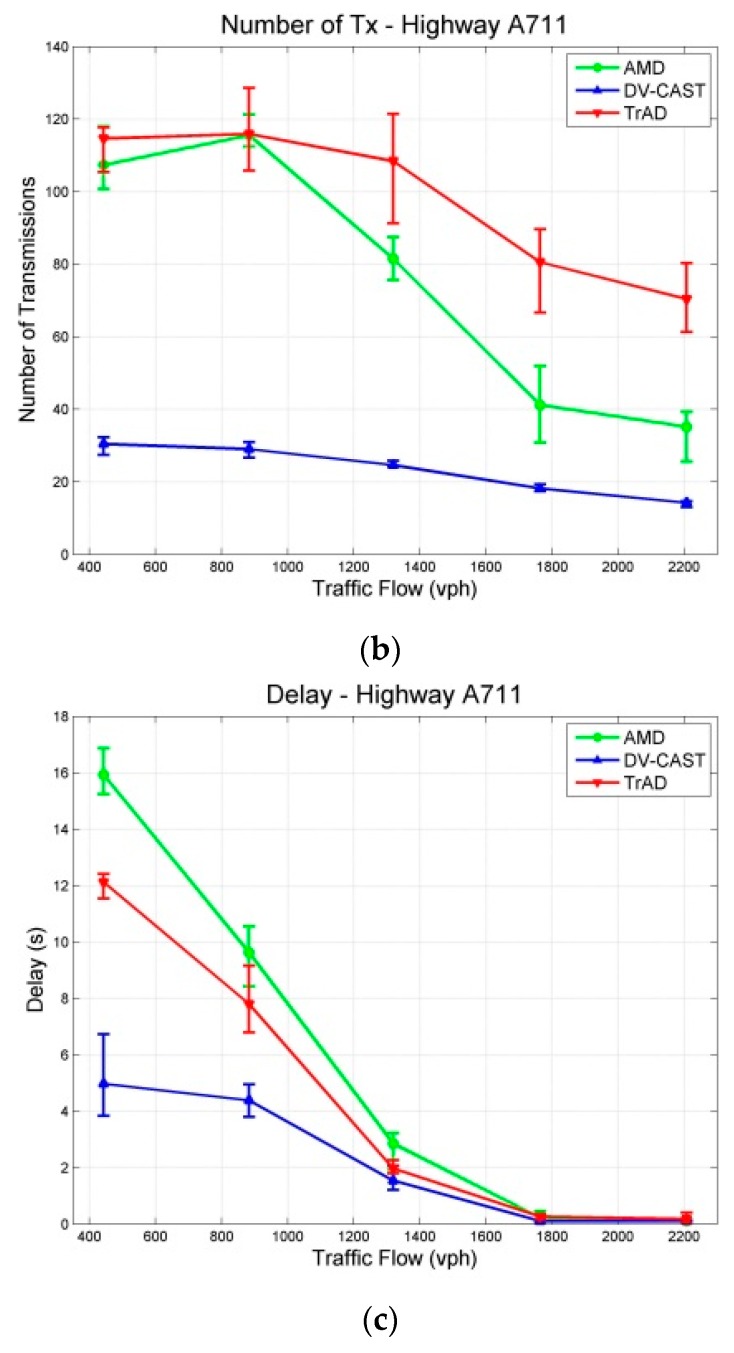
Results with a 95% confidence interval in Highway Scenarios: (**a**) PDR; (**b**) Number of Transmissions; (**c**) Delay.

To implement the highway map, a section of A711 highway located near Clermont-Ferrand airport (Aulnat, France) is modeled, as shown in Figure 11. It has two lanes in each direction and has 2 km length. The data message is created by the fixed source node at the west end of the highway and gathered by all vehicles driving in the ROI. To detect the *traffic flow*, induction loop detectors were implemented under each lane as highlighted by the red circles in Figure 11. The unit of traffic flow represents the number of vehicles passed the detection point per hour (vph). We set five levels of traffic flow that include 443 vph, 883 vph, 1319 vph, 1764 vph and 2207 vph, which increase linearly. The simulation duration of each traffic flow is 200 s and repeated 10 times with different random seeds. All the obtained simulation results with a 95% confidence interval are shown in Figure 12.

In the case of highway scenarios, AMD achieves high PDRs that are similar as those of TrAD, as shown in Figure 12a, due to the relatively simple two-dimensional road topology. The PDR of AMD exceeds 90% since 883 vph and finally reaches close to 100%. However, Figure 12a also highlights that the PDR of DV-CAST fails to reach 70% finally. The reason lies in that DV-CAST does not have the rescue mechanism for failed transmissions. Indeed, the transmitter vehicles forward the data message only once, *i.e.*, one-shot deal. Theoretically, the operation principle of DV-CAST guarantees a high data coverage on the highway. However, if a transmission fails, there is no mechanism to trigger the retransmission again. It is possible that the consecutive transmissions would fail due to fading or collisions. Therefore, if a transmission is broken at the middle forwarder, the vehicles in the rear of this forwarder cannot receive the data message.

In addition, TrAD uses the message list in the beacon to detect data messages needed by neighbors, so that the failed transmission can be rescued in the next beaconing process. Since the PDR values of TrAD and AMD are close, it makes sense to compare their number of transmissions and delay, as shown in Figure 12b,c. AMD costs less transmissions than TrAD in dense networks, which is consistent with the result of experiments in [7]. However, the delay of TrAD is lower than the one of AMD. This means that the extra transmissions of TrAD would come from the SCF mechanism in one-hop broadcasting, especially at the border of the ROI. TrAD always employs the breaker going out of the ROI to load data messages to the vehicles just coming into the ROI. That is why the PDR of TrAD gains the advantage over AMD at most of vehicular densities, e.g., 443 vph, 1319 vph and 1764 vph. Although the number of transmissions and the delay of DV-CAST are the lowest, its overall performance is still the worst, because the low transmissions and the low delay exactly benefit from the low PDR.

### 4.4. GPS Drift

Some approaches of TrAD are based on vehicle position. However, the GPS system is usually not accurate, particularly in downtowns. This would be affected by several impact factors, such as satellite position and number, signal attenuation and clocking errors. In particular, in urban scenarios, high towers could obstruct the GPS signal. The average deviation of standard GPS is up to 30 m [36]. Therefore, we investigated the GPS drift in terms of data dissemination speed that was defined at the beginning of this section. Error deviations have been injected into the mobility module of Veins framework. We choose five options of error deviations, *i.e.*, 0 m, 25 m, 50 m, 75 m and 100 m. The 0 m deviation means the perfect positioning, and other instantaneous deviation values are selected between the plus and minus interval following uniform distribution. For the instance of 25 m deviation, the *x* axis and the *y* axis of deviation values are obtained randomly between −25 and 25 and added to the original correct coordinate. To evaluate the impact of GPS drift on the performance of protocols, the moderate vehicle density, *i.e.*, 100 v/km^2^, is used in the Clermont-Ferrand and Manhattan scenarios with traffic route 1 (uniform).

**Figure 13:**
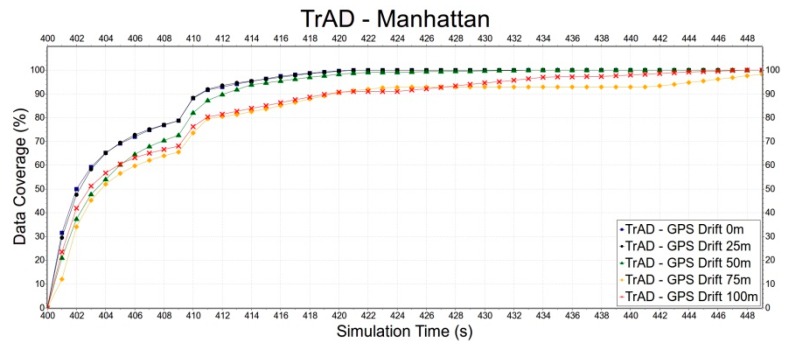
The impact of GPS drift on TrAD in the Manhattan scenario.

**Figure 14:**
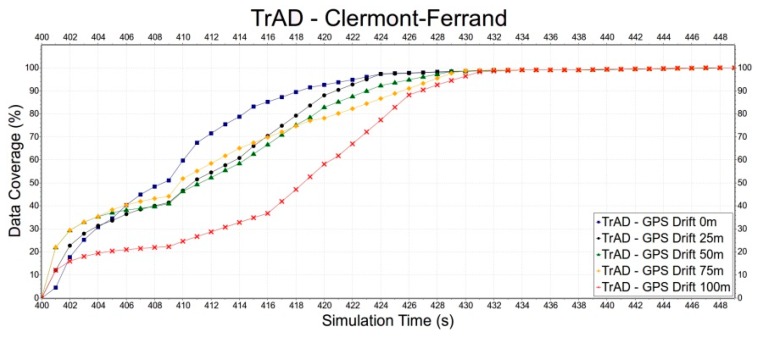
The impact of GPS drift on TrAD in Clermont-Ferrand scenario.

First, the robustness of TrAD to GPS drift is tested in the Clermont-Ferrand and Manhattan scenarios, as shown in Figure 13 and Figure 14. In Figure 13, we can observe that thanks to the regular road topology and building obstacles TrAD maintains a relatively good performance in the Manhattan scenarios except 75 m and 100 m positioning deviations. However, in the case of Clermont-Ferrand, Figure 14 presents that the data dissemination speed is slower than the one of the Manhattan scenario due to the complex nature of the traffic environment. The 25 m, 50 m and 75 m error deviations impact the performance of TrAD, but the data dissemination speed of these deviations still achieves a moderate performance. The result of 100 m deviation is the worst, but its data coverage also reaches 98.3% taking 31 s after the data dissemination has been started.

**Figure 15:**
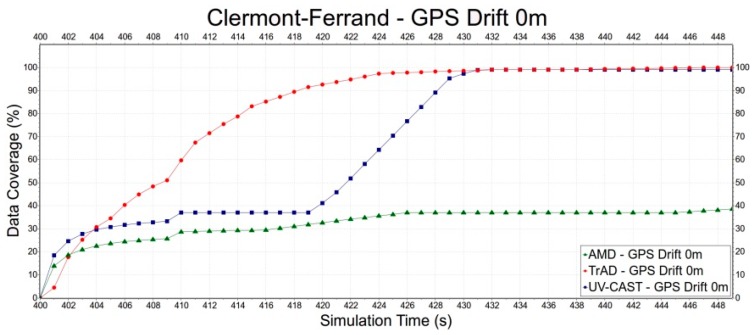
The data dissemination speed of TrAD, AMD and UV-CAST with perfect GPS.

**Figure 16:**
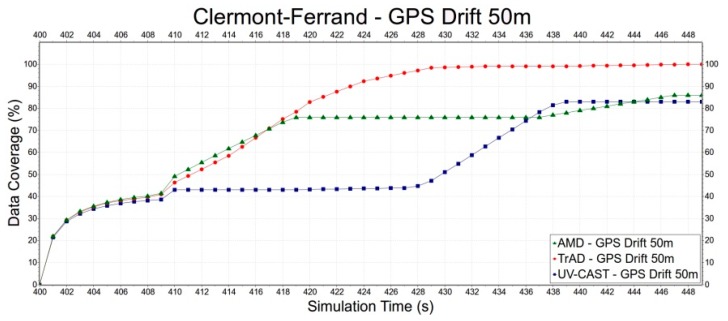
The data dissemination speed of TrAD, AMD and UV-CAST with 50 m GPS drift.

**Figure 17:**
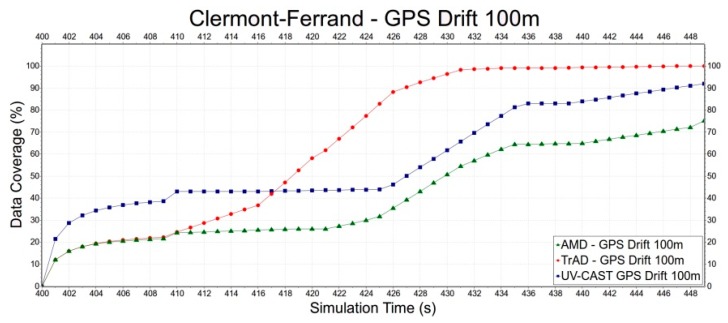
The data dissemination speed of TrAD, AMD and UV-CAST with 100 m GPS drift.

Second, we select the Clermont-Ferrand scenario to evaluate the robustness of TrAD, AMD and UV-CAST to the GPS drift, since the Clermont-Ferrand scenario has more influence on TrAD than the Manhattan one. Error deviations include 0 m, 50 m and 100 m, as shown in Figure 15, Figure 16 and Figure 17. In Figure 15, the data coverage of TrAD increases rapidly to 100% while the data coverage of UV-CAST climbs up much slower than TrAD and finally reaches 99.1%. In Figure 16, TrAD still keeps a good upward trend and reaches 100% data coverage. It is interesting that, in this case, the GPS drift limits the performance of UV-CAST and let its dissemination speed be lower than the one of AMD at most of time. Finally, UV-CAST and AMD achieve 86% and 83% data coverage, respectively. In Figure 17, although the large positioning deviation hurts TrAD very badly, the data coverage of TrAD still exceeds the other two protocols 17 s after data dissemination starts and achieves 100% at the end. In this simulation, the dissemination speed of UV-CAST is always better than AMD, which is not the case in Figure 16. Finally, UV-CAST and AMD achieve 92% and 75.1% data coverage, respectively. The abovementioned observations reveal that the random GPS drift will cause unreliable results in the protocols with less robustness. Although, for TrAD, the increasing slope of its data dissemination speed gradually decreases with the increasing of error deviations, it always takes the shortest time to reach 100% data coverage.

## 5. Conclusions and Future Work

In this paper, a new adaptive data dissemination protocol named TrAD is proposed for both highway and urban scenarios, taking into account both road traffic and network traffic status. The realistic simulation results show that TrAD outperforms three well-known reference data dissemination protocols in both highway and urban scenarios. On the one hand, TrAD is flexible enough to adapt to irregular road topology and considers both road traffic and network traffic status for the transmission delay-contention scheme and sorting technique. On the other hand, TrAD uses double broadcast suppression techniques to limit data message transmissions. This technique not only performs the broadcast suppression technique in a well-connected network, but also limits the rebroadcast of SCF-agents in disconnected networks. Consequently, TrAD only needs a moderate amount of data message transmissions to achieve high data coverage with low delay. Furthermore, TrAD can alleviate well the impact of GPS drift up to 75 m in a complex traffic environment, e.g., the Clermont-Ferrand scenario. In conclusion, TrAD provides good realistic simulation results in terms of PDR, data message transmission and delay, but our next challenge is to implement TrAD in vehicles and to evaluate its field test performance. Moreover, on the one hand, we will investigate the security and privacy issues to improve the TrAD protocol and we know that this will be a big challenge to overcome [37,38]. On the other hand, how to implement a standard large scale field test of IVC protocol is also still an open research issue.

## Figures and Tables

**Table 1:** Simulation setting.

**Physical Layer**	Frequency band	5.89 GHz
	Bandwidth	10 MHz
	Tx power	300 mW
	Receiver sensitivity	−100 dBm
	FSPL exponent α	3.0
	Thermal noise	−110 dBm
	Radio range (Friis)	~366 m
**Link Layer**	Bit rate	6 Mbit/s
	CW	[15, 1023]
	Slot time	13 μs
	SIFS	32 μs
	DIFS	58 μs
**Data Broadcasting**	Broadcast frequency	0.5 Hz
	Data size	2312 bytes
**Beaconing**	Beacon frequency	1 Hz
	Beacon size	378 bytes
	Message list entries	40
**TrAD**	ti	5 ms
	α	10°
	max.#Neighbor	25
	max.RadioRange	~366 m
**AMD**	st	5 ms
	${\text{ts}}_{d}$	1
	${\text{AD}}_{\text{ij}}$	DIFS
**UV-CAST**	$\tau_{\text{max }}$	500 ms
**DV-CAST**	st	5 ms
	$N_{st}$	5
	WAIT I	120 s
	WAIT II	120 s

**Table 2:** Statistics of maps.

City Map	Clermont-Ferrand	Manhattan
Total lanes	366	166
Total junctions	137	86
Avg. street length	97.39	151.45
Avg. lanes/street	1.62	1.11
